# fMRI of Working Memory Impairment after Recovery from Subarachnoid Hemorrhage

**DOI:** 10.3389/fneur.2013.00179

**Published:** 2013-11-06

**Authors:** Timothy M. Ellmore, Fiona Rohlffs, Faraz Khursheed

**Affiliations:** ^1^Department of Psychology and Program in Behavioral and Cognitive Neuroscience, The City College of New York, New York, NY, USA; ^2^Department of Vascular Medicine, University Hospital Eppendorf, Hamburg, Germany; ^3^Department of Neurology, Louisiana State University Health Science Center, New Orleans, LA, USA

**Keywords:** aneurysm rupture, verbal memory, phonological loop, mixed-effects multilevel analysis, stroke

## Abstract

Recovery from aneurysmal subarachnoid hemorrhage (SAH) is often incomplete and accompanied by subtle but persistent cognitive deficits. Previous neuropsychological reports indicate these deficits include most prominently memory impairment, with working memory particularly affected. The neural basis of these memory deficits remains unknown and unexplored by functional magnetic resonance imaging (fMRI). In the present study, patients who experienced (SAH) underwent fMRI during the performance of a verbal working memory paradigm. Behavioral results indicated a subtle but statistically significant impairment relative to healthy subjects in working memory performance accuracy, which was accompanied by relatively increased blood-oxygen level dependent signal in widespread left and right hemisphere cortical areas during periods of encoding, maintenance, and retrieval. Activity increases remained after factoring out inter-individual differences in age and task performance, and included most notably left hemisphere regions associated with phonological loop processing, bilateral sensorimotor regions, and right hemisphere dorsolateral prefrontal cortex. We conclude that deficits in verbal working memory following recovery from (SAH) are accompanied by widespread differences in hemodynamic correlates of neural activity. These differences are discussed with respect to the immediate and delayed focal and global brain damage that can occur following (SAH), and the possibility that this damage induces subcortical disconnection and subsequent decreased efficiency in neural processing.

## Introduction

Subarachnoid hemorrhage (SAH) due to aneurysm rupture accounts for about five percent of all strokes and affects approximately 30,000 individuals annually in the United States (1–3). In contrast to other types of stroke, the incidence of aneurysmal SAH has not declined in the last few decades (3). This is likely due to high prevalence of clinically silent intracranial aneurysms in otherwise healthy individuals (4–6).

Traditionally, aneurysmal SAH has been associated with high mortality and morbidity, but the last few decades have seen a tremendous improvement in overall care of such patients. Associated mortality has fallen by about 50% in the last two decades with case fatality rates improving at 0.9% every year. Interestingly, this has not been associated with a rebound increase in the proportion of survivors with severe neurological disabilities (3). Still, about two thirds of SAH survivors will experience some long-term neurological deficit (7).

Although most published outcome data from survivors of aneurysmal SAH place patients with mild disabilities in a “good recovery” category (3), several studies have demonstrated that these patients suffer from varying degrees of cognitive impairment (8–13). Despite the increasing prevalence of such “high functioning” survivors, more research is critically needed to understand how specific cognitive deficits following recovery form SAH relate to specific neurobiological changes so that rehabilitation strategies can be improved.

The physical, economic, and emotional toll of SAH is so dramatic (14–16) because the median age of aneurysmal hemorrhagic stroke is younger than for ischemic stroke and therefore encompasses more years of productive potential and higher total costs of rehabilitation. If the patient survives, recovery is often long and a number of neuropsychological studies indicate a spectrum of subtle but persistent cognitive problems that affect daily living and return to work (17–23). Of particular interest are reports in SAH patients that memory function is often affected (24–26), with verbal memory (27) and aspects of the phonological store particularly impaired (28).

There are several questions that remain to be answered about impaired memory function in SAH patients. From a behavioral perspective, it remains to be determined what aspects of short-term memory, particularly working memory (25), are most affected. Do patients experience deficits in the encoding, maintenance, or retrieval of information? Are these differences reflected in the overall performance accuracy of working memory trials, or in the speed with which working memory computations are carried out? From a biological perspective, the neural basis of memory impairment in these patients is not at all understood. Are behavioral impairments in SAH patients accompanied by increased or decreased activity? If so, how are activity differences related to performance and age, and are the differences limited to the encoding, maintenance, or retrieval operations of working memory? A challenge in answering brain-behavior questions like these has been that SAH patients, particularly those treated neurosurgically with implanted aneurysm clips, are difficult to image in a high field MRI environment, unless they have been implanted with MRI-safe titanium clips that produce minimal artifact (29).

In the present study, we undertook an exploratory behavioral and neuroimaging investigation of SAH patients with two main objectives. First, we examined verbal working memory using a well-validated task paradigm in order to measure the accuracy and speed of working memory processing. Second, using this task we examined using 3 T functional magnetic resonance imaging (fMRI) the blood oxygenation level dependent (BOLD) activity during the three temporal phases of working memory encoding, maintenance, and retrieval. Differences between patients and controls in the fMRI-BOLD activity patterns and their covariation with measures of age and performance are presented, followed by discussion and some preliminary conclusions regarding the behavioral and neural correlates of verbal memory impairment in SAH.

## Materials and Methods

### Participants

Eleven patients who experienced SAH participated in this study. Detailed demographic and clinical characteristics for each patient are summarized in Table 1. Ten healthy participants (5 males, mean age 27.3, range 20–55 years old, 1 left handed) with no history of cerebrovascular or psychiatric diagnoses served as controls. Each participant provided written informed consent in accordance with a protocol approved by the local institutional review board for ethical research and protection of human subjects. Each participant completed a single 90 minute magnetic resonance imaging session that included the performance of a working memory task during the collection of functional imaging data.

**Table 1:** **Patient demographics and clinical characteristics**.

Patient	1	2	3	4	5	6	7	8	9	10	11
**DEMOGRAPHICS**											
Age	58	58	49	49	62	52	67	36	57	50	44
Sex	F	F	F	F	F	F	F	F	F	M	M
Handedness	R	R	R	R	R	R	L	R	R	R	R
Hunt and Hess grade	4	2	1	2	3	1	2	4	2	1	2
Fisher’s grade	4	2	2	3	4	1	3	4	2	1	1
SAH distribution	Global	Focal (R occipital)	Global	Global	Global	Global	Global	Global	Global	Global	Global
SAH etiology	L PICA aneurysm	Undetermined	Fenestrated R MCA	L Ant Choroidal A^c^	ACoM	Basilar tip aneurysm	Undetermined	L ACA	R PCOM	R PCOM	R ACA
Aneurysm location	L PICA, R ICA, L ICA	NA	NA	L Ant Choroidal A, L PCOM, 2 ACoM	ACoM	Basilar tip aneurysm	NA	L ACA	R PCOM, L PCOM(diagnosed 1 year after)	R PCOM, 2 R MCA, L PCOM	R ACA
Intraparenchymal component	Yes	No	No	No	No	No	No	Yes	No	No	No
IVH	Yes	No	No	No	No	No	No	Yes	No	No	No
Focal neurological deficits	Yes	No	No	No	No	No	No	Yes	No	No	No
Vasospasm	Clinically significant^a^	No	Clinically significant^a^	Clinically significant^a^	Clinically significant^a^	No	No	Clinically significant^a^	No	No	No
**TREATMENT**											
GDC coiling^b^	Yes	No	No	Yes	Yes	Yes	No	Yes	Yes	No	Yes
Craniotomy	Yes	No	No	Yes	Yes	No	No	Yes	Yes	Yes	No
Approach	L Pterional	NA	NA	L Pterional, Rectus gyrus resection	L Pterional	NA	NA	L Pterional	L Pterional	R Pterional, L Pterional	NA
Operative findings	NAD	NA	NA	Hemosiderin deposition	Significant arachnoidal scarring and hemosiderin deposition	NA	NA	Diffuse subrachnoid blood	Arachnoidal scarring, hemosiderin deposition	Subarachnoid hemorrhage	NA
Coiling	Yes	NA	NA	Yes	Yes	Yes	NA	Yes	Yes	NA	Yes
Craniotomy and clipping	Yes	NA	NA	Yes	Yes	NA	NA	Yes	Yes	Yes	NA
Time between SAH and surgery	75 days	NA	NA	46 days	315 days	NA	NA	15 days	4 months (no sig)	1 day	0 days
**CLIP SPECIFICATIONS**											
Number of clips	0	0	0	3	1	0	0	1	1	4	NA
Manufacturer	NA	NA	NA	Aesculap	Aesculap	NA	NA	Aesculap	Aesculap	Aesculap	NA
Composition	NA	NA	NA	Titanium	Titanium	NA	NA	Titanium	Titanium	Titanium	NA
Size	NA	NA	NA		7 mm	NA	NA	11 mm			NA
Weight	NA	NA	NA		0.08 gm	NA	NA	0.19 gm			NA
Width	NA	NA	NA		0.95 mm	NA	NA	1.35 mm			NA
Shape	NA	NA	NA		Straight	NA	NA	Straight			NA
Model	NA	NA	NA	FT722T, FT942T, FT744T	FT720T	NA	NA	FT760T	FT752T	FT742T, FT700T, FT754T, FT720T (L PCOM)	NA
**fMRI**											
Time between SAH and fMRI	5 months	2.5 months	6 months	7 months	30 months	2 months	120 months	16 months	17 months	1.5 months	1 month

^a^Requiring endovascular intervention, -ve if NO angiographic evidence of vasospasm.

^b^Coils used are Micrus, EV3, Boston Scientific.

^c^Last branch of ICA.

### Working memory task

Each participant completed eight blocks of a variant of a Sternberg (30) verbal working memory task that we have previously used successfully during other patient neuroimaging studies (31). During this task, participants were presented visually in the scanner bore with a sequence of four letter strings. Each white letter appeared for 1 s on a black background, one at a time followed by a 1 s black screen, during an encoding period of 8 s. The encoding period was followed by a maintenance or delay period of 6 s during which the participant was required to hold in memory the four previously presented letters. Each sequence of letters did not spell a word, and participants were not dissuaded from repeating the sequences to themselves during the maintenance period in order to maintain successfully the information online. After the maintenance period, a probe letter was presented for 1 s, and the participant was required to indicate within a time limit of four additional seconds with a button press whether or not this letter was one of the previously four presented letter strings. The 14 s temporal sequence of encoding, maintenance, and retrieval periods relative to the fMRI data collection can be seen in the colored vertical bars that appear in Figures 7C,D. Participants did not receive any feedback during scanning about whether their answer on each probe trial was correct or incorrect.

An 18 s control period was placed between the sequence of eight working memory trial blocks to allow for fMRI signal to return to baseline before a new encoding period started. To control for visuomotor processing unrelated to working memory processing, the baseline period consisted of change detection in the background color of sequentially presented crosses. A total of seven crosses appeared during each baseline period with six appearing on a white background and with one appearing randomly on a green background. Participants were instructed to press a button when a cross on the green background appeared during the center fixation.

### Image acquisition

Brain scans were obtained with a 3 T Philips Intera scanner (Philips Medical Systems, Bothell, WA, USA) equipped with an eight-channel head coil (SENSE acquisition). A high-resolution 3D T1-weighted magnetization prepared rapid acquisition turbo field sequence was acquired (TR/TE = 8.4/3.9 ms; flip angle = 8°; matrix size = 256 × 256; FOV = 240 mm; slice thickness = 1.0 mm sagittal slices). During working memory task performance, functional images were obtained with a gradient-recalled echo-planar imaging sequence sensitive to blood-oxygen level dependent contrast (TR/TE = 2000/30 ms; flip angle = 90°; matrix size = 80 × 80; field of view = 220 mm; 3 mm thick axial slices, 136 dynamics).

### Image analysis

All image analysis was performed using tools in the freely available AFNI software package (32, 33). For each participant, each fMRI volume was aligned to the skull-stripped T1-weighted MRI volume. An affine transform of the T1-weighted volume to MNI space was then applied to each fMRI volume. Then voxelwise mixed-effects multilevel (MEMA) within- and between-group analyses were conducted on spatially normalized fMRI volumes by comparing encoding, maintenance, and retrieval periods with the baseline control periods. The MEMA group analysis approach (34) employed here has several advantages compared to traditional fMRI group analyses. It incorporates variability across subjects and precise estimates of each effect of interest (i.e., each working memory processing period) from individual subject analyses, leading to higher statistical power especially when there are outliers (non-Gaussian distributions) and conventional variance assumptions do not hold. The MEMA approach also allows for incorporation of subject-specific covariates, which in the present study included age and working memory accuracy (percent correct).

For each working memory period (encoding, maintenance, retrieval) relative to the control baseline, voxelwise *t*-statistic maps were computed and thresholded with recommended (34) parameters (*p* < 0.05, corrected) for each group of controls and patients (within-group analyses). MEMA t-maps were also computed for the difference between patients and controls for each processing period (between-group analyses). Finally, t-maps were computed summarizing at each voxel how the BOLD-fMRI signal fluctuated differently as a function of age and performance accuracy between the patients and controls. The within- and between-group activity and covariate maps were projected in different colors as a function of processing period to pial and white/gray matter border surface reconstructions of the TT_N27 brain in MNI space for visualization of whole-brain differences.

## Results

### Behavior

**Figure 1:**
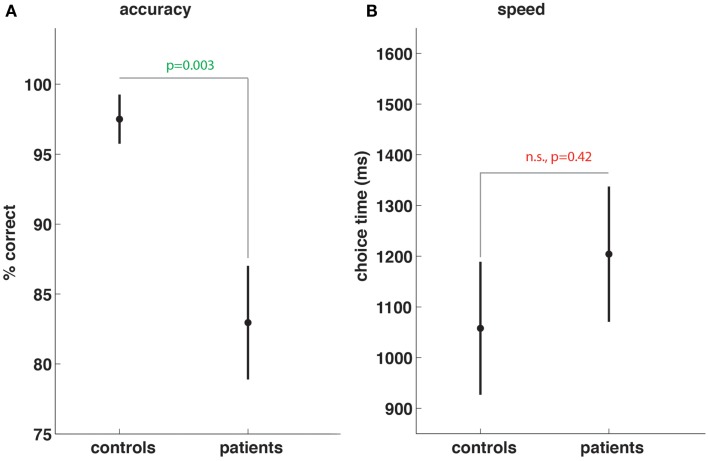
**Subarachnoid hemorrhage patients show working memory task impairment**. Compared to controls, SAH patients show significant reductions in working memory task accuracy **(A)** but no significant differences in speed of choices made during memory trials **(B)**.

Patients performed significantly worse (percent correct 82.9 vs. 97.5, *p* < 0.003, Figure 1A) in terms of working memory task accuracy. While the time taken by patients was longer (1204 vs. 1058 ms, Figure 1B) to decide whether the probe stimulus at retrieval was or was not one of the four stimuli previously presented during the encoding period, the difference was not significant (*p* = 0.42).

### Imaging

**Figure 2:**
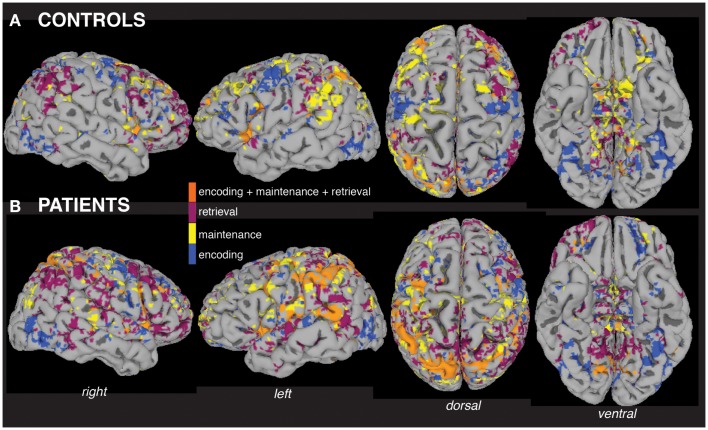
**Within-group analyses show distributed fMRI activity across working memory task periods**. Separate mixed-effects model analyses indicate significant activity (*p* < 0.05) across working memory encoding, maintenance (delay period), and retrieval task periods in the group of patients **(A)** and the group of controls **(B)**, including areas activated during all three periods (orange). The group maps are displayed for ease of comparison on a single subject healthy control brain surface, the TT_N27 brain model, which is distributed by the Montreal Neurological Institute.

Visualization of within-group MEMA analyses on the cortical surface revealed widespread and significant (*p* < 0.05, corrected) BOLD-fMRI activity across all working memory processing periods (Figure 2) relative to baseline. A side-by-side visual comparison indicated that task activity was more widespread in patients (Figure 2B) compared to controls (Figure 2A).

**Figure 3:**
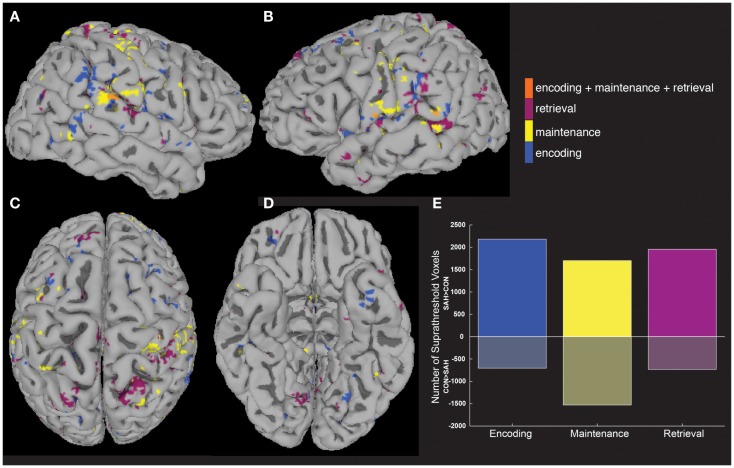
**Between-group analyses show greater activity in patients compared to controls**. A between-group analysis showed significantly greater (*p* < 0.05) fMRI activity in patients vs. controls across widespread brain areas **(A–D)** and during all three task periods **(E)**.

Increased activity in patients was tested quantitatively in a between-group analysis (Figures 3A–D), with patients showing more voxels activated compared to controls in each of the three task periods (Figure 3E). The highest number of greater task-activated voxels in patients was during the encoding period (Figure 3E, blue bar).

**Figure 4:**
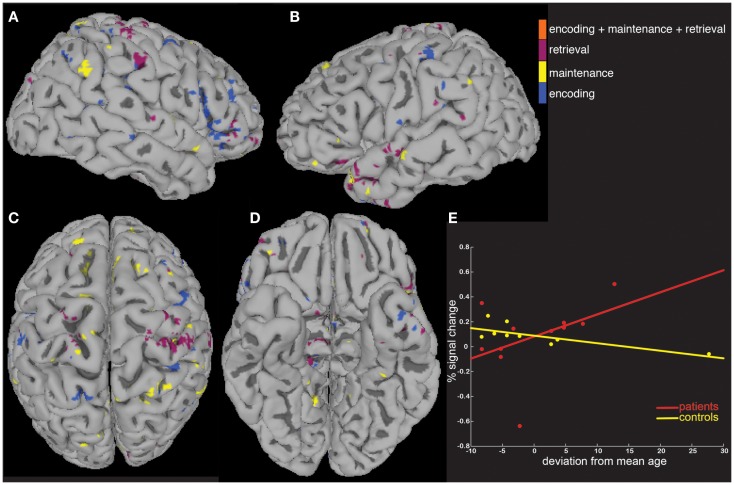
**A covariate interaction analysis shows regions that differ as a function of age**. Several regions **(A–D)** exhibited fMRI activity that covaried significantly (*p* < 0.05) differently between patients and controls as a function of age **(E)**.

**Figure 5:**
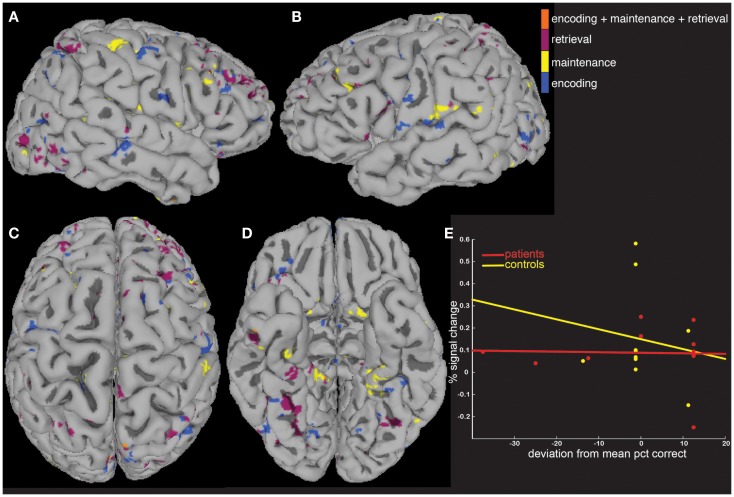
**A covariate interaction analysis shows regions that differ as a function of working memory accuracy**. Several regions **(A–D)** exhibited fMRI activity that covaried significantly (*p* < 0.05) differently between patients and controls as a function of working memory accuracy **(E)**.

We next tested the hypothesis that the greater activity elicited in patients could be explained by signal that covaried differently as a function of age and task performance. To do this, we computed MEMA age and percent correct covariate t-maps, and displayed significant BOLD-fMRI changes that differed between groups as a function of age (Figure 4) and performance (Figure 5). These analyses highlighted regions where signal differed differently between groups as a function of the covariate, and no constraint was placed on the direction of the difference. Averaged *across all regions and task periods* that were found to differ significantly between groups as a function of the age covariate, patients tended to exhibit BOLD-fMRI changes that increased with age while controls showed a decreasing pattern (Figure 4E). For the regions that differed between groups as a function of task accuracy, patients showed a mostly flat relationship while controls showed an average decrease as a function of increasing performance (Figure 5E).

**Figure 6:**
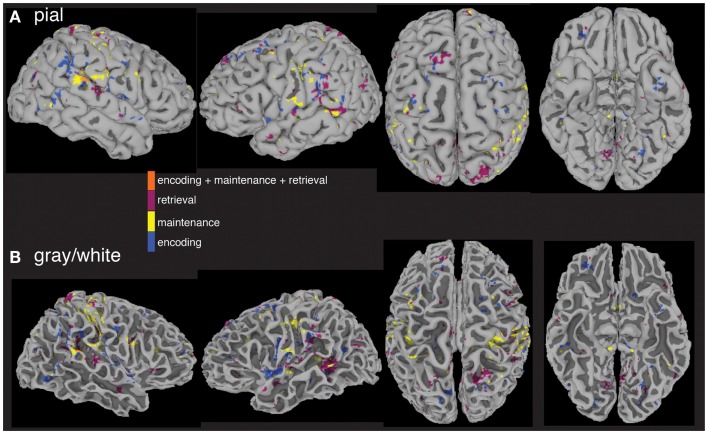
**Between-group fMRI activity differences masked by age and accuracy covariate interactions**. When significant age and accuracy interaction differences are subtracted from the between-group analysis, widespread regional increases (*p* < 0.05) in activity in patients vs. controls across all three task periods remain. Masked differences are shown on a pial **(A)** and gray/white border **(B)** surface reconstruction of the TT_N27 brain in MNI space.

The last question we asked using the computed MEMA maps was how much overlap existed between the age and performance covariate maps and the between-group difference map. Do the age (Figure 4) and performance (Figure 5) activity differences encompass the same regions found to differ in terms of total activity between the groups (Figure 3)? We multiplied a mask (consisting of ones) of thresholded between-group differences (Figure 3) by a mask (consisting of zeros) of the summed thresholded age and performance covariate maps to produce a map of between-group differences that cannot be explained by the age- and performance-related changes (Figure 6). Visualization of the resultant masked between-group activity on pial (Figure 6A) and gray/white border (Figure 6B) surfaces revealed several clusters of activity differences in both hemispheres (Table 2).

**Table 2:** **Between-group differences (SAH > CON)**.

Task period	No. voxels in cluster	Hemisphere	Cluster center of mass coordinate (MNI)	Brain region^1^
Encoding	358	Left	−35, −5, 6	Insula
	266	Right	48, −11, 22	Rolandic operculum
	169	Left	−50, −11, 23	Postcentral gyrus
	159	Left	−40, −54, 9	Middle temporal gyrus
	155	Left	−16, −34, 39	Superior parietal (5Ci)
	101	Right	26, 44, 15	Superior frontal gyrus
	96	Right	9, −57, 45	Precuneus
	90	Right	58, −49, 27	Supramarginal gyrus
	74	Left	−52, −30, 25	Supramarginal gyrus
	52	Right	45, −64, −12	Inferior temporal gyrus
Maintenance	685	Right	36, −30, 47	Postcentral gyrus
	198	Left	−47, −23, 40	Postcentral gyrus
	181	Right	23, −44, 13	Hippocampus
	48	Left	−27, −50, 8	Precuneus
	45	Left	−31, −45, −1	Parahippocampal gyrus
	45	Right	57, −2, 28	Precentral gyrus
	30	Left	−36, −3, 55	Middle frontal gyrus
	25	Left	−8, −23, 49	Middle cingulate gyrus
	24	Right	42, 26, 19	Inferior frontal gyrus
	24	Right	61, −24, 25	Supramarginal gyrus
Retrieval	237	Left	−54, −44, 0	Middle temporal gyrus
	140	Right	47, −41, 24	Supramarginal gyrus
	136	Right	16, −57, 66	Superior parietal lobule
	129	Right	41, −23, 12	Heschl’s gyrus
	112	Left	−4, −66, 19	Calcarine
	98	Right	43, 6, 30	Inferior frontal gyrus
	85	Right	48, −28, 33	Supramarginal gyrus
	78	Right	44, 23, 8	Inferior frontal gyrus
	77	Right	8, −66, −15	Cerebellar vermis
	76	Right	−47, −16, 5	Superior temporal gyrus

^1^based on cluster center of mass coordinate referenced to Eickhoff–Zilles probability maps.

**Figure 7:**
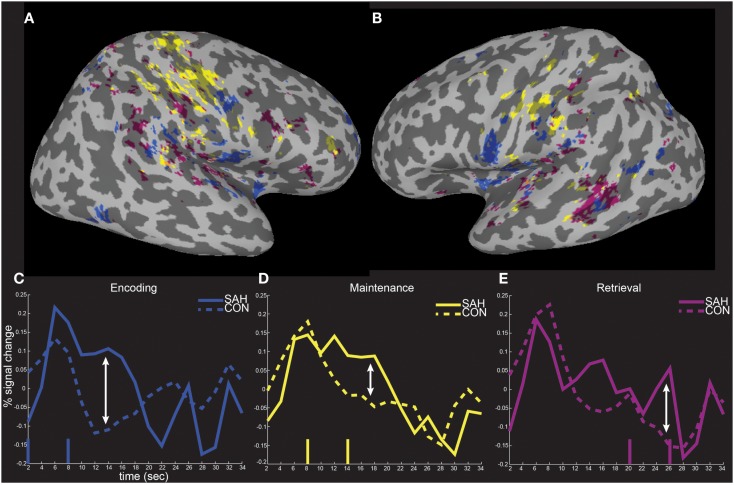
**Spatial localization of fMRI activity differences on inflated surface representations**. The fMRI activity increases in patients from Figure 6 is shown on right **(A)** and left **(B)** hemisphere inflated surface representations of the TT_N27 brain. Average temporal responses within each of the significantly differently activated task periods are shown relative to the start and end (vertical lines) of the encoding **(C)**, maintenance **(D)**, and retrieval **(E)** periods.

The spatial distributions of the masked activity differences were further investigated by inflating painted gray/white border reconstructions (Figure 7). The most prominent remaining activity differences were clustered near left hemisphere inferior frontal and precentral gyri (for encoding, blue, Figure 7B), left middle temporal gyrus (for retrieval, purple, Figure 7B), and in bilateral pre and postcentral somatosensory regions (for maintenance, yellow, Figures 7A,B). Spatial averages of BOLD-fMRI activity from regions highlighted in Figures 7A,B relative to the start and end of the encoding, maintenance, and retrieval periods are displayed in Figures 7C–E respectively (note lagged hemodynamic time courses).

## Discussion

In the present study, we report results from a combined behavioral and functional MRI study following recovery from SAH, a type of stroke that causes complex immediate and delayed secondary brain damage. Our study was motivated by several reports indicating cognitive deficits, including most prominently memory dysfunction, that persist after recovery from SAH and that severely impact quality of life (3, 8, 10, 35–37). The neural basis for these cognitive deficits after SAH remains poorly understood. While structural MRI studies have been conducted to understand how brain atrophy and lesion location relates to SAH cognitive dysfunction (38–42), to our knowledge the present study is the first to employ task-based fMRI to investigate the neural basis of memory in SAH patients.

Behavioral results obtained from our verbal working memory paradigm indicated that patients were significantly impaired relative to healthy subjects on retrieval accuracy, that is, determining whether or not a probe letter was presented previously during an encoding period. We used a relatively high load of four letters to be encoded, which according to our behavioral results and post-assessment debriefing was easy for healthy subjects but was cognitively challenging for the SAH patients. The majority of patients studied reported problems with memory and daily activities requiring memory, and these subjective reports were borne out by the objective accuracy differences obtained using the Sternberg paradigm. However, the patients’ retrieval time to decide during the probe trials was not significantly longer. These results are compatible with a deficit at stimulus encoding rather than at retrieval, since if all stimuli were encoded and stored properly their accuracy would be on par with controls, but their retrieval time on probe trials would be longer because of a difficulty in accessing the stored representations. Although our results are compatible with this scenario, it is too early to rule out other behavioral hypotheses, especially since other studies of working memory following SAH have indicated a problem with manipulating information in working memory rather than storing information without manipulation processing (25).

The neuroimaging findings of the present study paint a complex picture of the neural basis of memory deficits in SAH patients. As there have been no previous investigations using fMRI of cognitive function after SAH, it was unclear at the outset whether memory dysfunction would be accompanied by reduced or increased activation. The between-group MEMA analysis clearly showed increased fMRI activation in SAH patients relative to controls during all three working memory periods. The greatest difference, in terms of numbers of voxels activated, occurred during the encoding period. The increased activity in the SAH patients compared to controls is reminiscent of other fMRI studies designed to map the neural correlates of cognitive efficiency (43), where results suggest that a critical determinant of individual differences is the efficiency of interactions among brain regions, with impaired (or slower) subjects requiring more executive control, and therefore more brain activity, compared to healthy (or faster) individuals in order to perform successfully. More executive control implies greater activity in SAH patients in frontal regions, and our results clearly show increased activity in both left and right frontal cortex suggesting perhaps greater executive control during working memory processing (44).

The pattern of increased fMRI activation during encoding and retrieval in SAH patients, included left inferior frontal regions, part of the insula, the left middle temporal gyrus/superior temporal sulcus, and supramarginal parietal regions (Figures 7A–D, blue and purple). These brain areas have been suggested to constitute in part the functional anatomy of the phonological loop (45), which Baddeley theorizes is involved in retaining sequences of familiar words (46), and specifically with respect to the verbal working memory task performed here, letters. One idea to explain this pattern of results is that SAH patients have particular difficulty with the encoding and maintenance of these simple letters in memory, and the greater activity is a correlate of decreased efficiency and increased effort associated with underlying neural computations of the phonological loop. Increased activity associated with maintaining the encoded stimuli could be reflected in SAH patients by greater delay activity we found in bilateral sensorimotor regions (Figures 7A,B,D, yellow), which overlaps with previously reported dorsal and caudal regions implicated in memory and response preparation respectively (47).

The greater activity exhibited by SAH patients was widespread across cortex, and it encompassed all three working memory task periods. Originally we hypothesized that these widespread differences could be explained by BOLD-fMRI signal that changed differently between patients and healthy subjects as a function of age and overall performance. Yet when we quantified individual age and performance-related differences statistically, and masked regions showing these effects from the overall between-group difference map, several regions of greater activity in patients remained across several brain areas (Figures 6 and 7; Table 2). Future studies in larger groups of SAH patients need to be conducted in order to investigate the variables that contribute to these differences. An examination of the clinical characteristics (Table 1) reveals the challenges in studying these types of patients. The SAH distribution is often global and diffuse, and the origin of the SAH is sometimes undetermined in that in some patients there is evidence of SAH but the precise rupture site is not visible using angiographic techniques.

While some studies point to particular vulnerability of anterior basal forebrain damage for impacting memory function (48), other investigators have had difficulty in pinpointing robust relationships between rupture site, evidence of brain damage, and specific memory impairment (25), which suggests diffuse rather than focal pathology underlie the deficits. In addition to diffuse pathology, our sample’s clinical characteristics (Table 1) also highlight the variability in the type of secondary delayed brain damage following SAH. Due to our limited sample and the exploratory nature of this preliminary study, we cannot quantify reliably the separable contributions of vasospasm, hemosiderin deposition, arachnoidal scaring, hydrocephalus, retraction injury, and intraparenchymal damage to the behavioral deficits and BOLD-fMRI changes. The clinical heterogeneity of damage and diffuse pathology is ideal for future study using techniques like network analysis of structural and functional MRI data (49), which has shown promising in other patient groups for relating cognitive changes with aspects of diffuse brain pathology (50).

Another promising tool for gaining a deeper understanding of the neural basis of SAH memory deficits is diffusion MRI tractography, which allows for reconstruction of white matter fiber tracts near rupture locations. In a preliminary report of two SAH patients, one with an ACA and one with an ACoM rupture, tractography in regions around rupture locations, the putative sites of focal damage, was shown to identify the left uncinate fasciculus and inferior frontal occipital fasciculus (29). These tracts connect distant brain regions including anterior temporal lobe with inferior frontal lobe, and the occipital lobe with inferior frontal lobe and have been shown to be important for aspects of language (51) and working memory function (52). Another tract that may be vulnerable to aneurysms of the anterior circulatory system is the arcuate fasciculus, which mediates language processes (53–55) and passes superior-lateral to the insula and connects the inferior frontal region with middle temporal region, three regions that were more activated in our sample of SAH patients. Finally, ACoM rupture can affect the fornix, a tract which connects the hippocampus and is implicated in short-term learning (56) and working memory (57). Based on the demonstrated ability of tractography to reconstruct fibers of passage around anterior aneurysm rupture sites (29), it is unlikely that a complete disconnection of tracts causes cognitive deficits, but subtle changes in microstructural aspects of the white matter could impact the efficiency of neural communication possibly leading to increased task-related BOLD-fMRI activation, which is a hypothesis future diffusion imaging studies may test.

The difference in memory accuracy between the two study groups could also be influenced by the mean age difference between the two groups. While we quantified inter-individual differences in age between groups in the MEMA analyses, our control group was younger on average than the SAH group. Our main goal in recruiting healthy participants was to control as best as possible for the multiple variables of gender, handedness, and age, and at the same time obtain a group of subjects who did not have a history of cerebrovascular disease. It is true that our SAH group is older on average than our healthy control group, and this difference could be contributing to the poorer performance of the SAH group. Working memory has been reported to decline with age, but pronounced deficits usually manifest after age 60, an age that is older than the average age of our SAH group (58). One could argue that memory decrements would be expected to be significantly poorer for a group of SAH patients older (e.g., elderly) than the group we studied. But even that prediction is not entirely a given, as one recent comparison of elderly participants and young participants show equal levels of performance in verbal short-term memory tasks even after controlling for differences in sensory processing (59). Some argue that visual working memory binding is mostly intact in the elderly relatively to younger subjects, with specific decrements only arising in diseases such as Alzheimer’s (60). The SAH group studied here is younger than elderly, and included patients in the middle age to older middle age range.

During the course of normal aging, neuroimaging studies indicate that older adults often show a greater extent of brain activation (i.e., overactivation) compared to younger adults at similar levels of difficulty. An idea to explain this is the Compensation-Related Utilization of Neural Circuits Hypothesis (61) whereby older subjects need to recruit more neuronal resources at lower loads than younger adults leaving fewer resources for processing at higher loads. Predictions consistent with this hypothesis include higher activity for older subjects during low working memory load and lower activity for older subjects during high working memory load. Under this scenario, at the relatively high working memory load of four used in the present study, one might predict that if our findings were driven mostly by age, then the SAH group would show lower fMRI activity, not higher activity, compared to younger controls. To fully evaluate this hypothesis as an alternative explanation for our findings, we would have had to utilize a parametric design varying working memory load beyond four items, as recent work indicates this is the point at which older subject performance throughput saturates (62). One could argue that the pattern of increased brain activity detected by fMRI in our SAH patients is similar to studies of aging, Alzheimer’s disease, and related neurological diseases in which working memory is affected. There are data suggesting hyperactivity in subjects at risk for Alzheimer’s disease (63), and in subjects with mild cognitive impairment (64). But some of the data are conflicting, with Alzheimer’s patients showing hyperactivity in some brain areas during verbal working memory, and hypoactivity in other brain regions (65).

To conclude, the present study provides the first fMRI evidence of working memory impairment following recovery from SAH. The behavioral finding of subtle but significant impairment in task working memory task performance is accompanied by increased BOLD-fMRI activity across widespread brain areas and during the encoding, maintenance, and retrieval periods. Future investigations should compare larger samples of SAH patients to age-matched patients who have mild cognitive impairment, those at risk for Alzheimer’s disease, and those diagnosed with Alzheimer’s disease in order to identify with more specificity how the pattern of brain damage unique to SAH affects working memory, and to relate the altered functional activity profiles to underlying structural connectivity changes.

## Author Contributions

Designed the study: Timothy M. Ellmore; Collected the data: Timothy M. Ellmore, Fiona Rohlffs, Faraz Khursheed; Analyzed the data: Timothy M. Ellmore, Fiona Rohlffs, Faraz Khursheed; Wrote the paper: Timothy M. Ellmore, Faraz Khursheed.

## Conflict of Interest Statement

The authors declare that the research was conducted in the absence of any commercial or financial relationships that could be construed as a potential conflict of interest.
